# Evidence for Resident Memory T Cells in Rasmussen Encephalitis

**DOI:** 10.3389/fimmu.2016.00064

**Published:** 2016-02-23

**Authors:** Geoffrey C. Owens, Julia W. Chang, My N. Huynh, Thabiso Chirwa, Harry V. Vinters, Gary W. Mathern

**Affiliations:** ^1^Department of Neurosurgery, David Geffen School of Medicine at UCLA, Los Angeles, CA, USA; ^2^Intellectual and Developmental Disabilities Research Center, David Geffen School of Medicine at UCLA, Los Angeles, CA, USA; ^3^Department of Pathology and Laboratory Medicine, David Geffen School of Medicine at UCLA, Los Angeles, CA, USA; ^4^Department of Neurology, David Geffen School of Medicine at UCLA, Los Angeles, CA, USA; ^5^Brain Research Institute, David Geffen School of Medicine at UCLA, Los Angeles, CA, USA; ^6^Mattel Children’s Hospital, Los Angeles, CA, USA

**Keywords:** Rasmussen encephalitis, focal cortical dysplasia, resident memory T cells, alpha beta T cells, gamma delta T cells

## Abstract

Rasmussen encephalitis (RE) is a rare pediatric neuroinflammatory disease of unknown etiology characterized by intractable seizures, and progressive atrophy usually confined to one cerebral hemisphere. Surgical removal or disconnection of the affected cerebral hemisphere is currently the only intervention that effectively stops the seizures. Histopathological evaluation of resected brain tissue has shown that activated brain resident macrophages (microglia) and infiltrating T cells are involved in the inflammatory reaction. Here, we report that T cells isolated from seven RE brain surgery specimens express the resident memory T cell (T_RM_) marker CD103. CD103 was expressed by >50% of CD8^+^ αβ T cells and γδ T cells irrespective of the length of time from seizure onset to surgery, which ranged from 0.3 to 8.4 years. Only ~10% of CD4^+^ αβ were CD103^+^, which was consistent with the observation that few CD4^+^ T cells are found in RE brain parenchyma. Clusters of T cells in brain parenchyma, which are a characteristic of RE histopathology, stained for CD103. Less than 10% of T cells isolated from brain specimens from eight surgical cases of focal cortical dysplasia (FCD), a condition that is also characterized by intractable seizures, were CD103^+^. In contrast to the RE cases, the percent of CD103^+^ T cells increased with the length of time from seizure onset to surgery. In sections of brain tissue from the FCD cases, T cells were predominantly found around blood vessels, and did not stain for CD103. The presence of significant numbers of T_RM_ cells in RE brain irrespective of the length of time between clinical presentation and surgical intervention supports the conclusion that a cellular immune response to an as yet unidentified antigen(s) occurs at an early stage of the disease. Reactivated T_RM_ cells may contribute to disease progression.

## Introduction

Rasmussen encephalitis (RE) is a rare chronic neuroinflammatory disease that primarily affects young children (1, 2). In the acute stage of the disease, RE patients present with intractable partial (focal) seizures that may spread to the rest of the brain. Hyperintensity in T2/FLAIR magnetic resonance images usually in one cerebral hemisphere is indicative of inflammation and atrophy (3). The inflammation may spread through the affected cerebral hemisphere, but generally does not cross over to the contralateral hemisphere (3). In the residual stage of the disease, there is significant unilateral brain atrophy and permanent neurological deficits that may affect motor and sensory systems. Intravenous immunoglobulins and tacrolimus can slow the destruction of brain tissue, but cannot reverse the intractable seizures (4). Early treatment of a presumptive RE case with ganciclovir was reported to ameliorate the seizures (5). Ultimately, surgical removal or disconnection of the affected cerebral hemisphere is the only intervention that stops the seizures, but inevitably leaves the patient with some functional deficits. A better understanding of the inflammatory processes that occur in RE may lead to the development of alternative non-surgical treatments.

The inflammatory reaction in RE involves both activated brain resident macrophages (microglia) and T cells (6–9). Histopathological examination of resected brain tissue and brain biopsies show T cells in perivascular spaces, leptomeninges, and in small clusters scattered throughout the affected grey and white matter (7, 9). Here, we report that many of the T cells in the parenchyma of affected RE brain tissue at the time of surgery are resident memory T cells (T_RM_) as evidenced by the expression of CD103, the αE integrin subunit. T_RM_ cells are a distinct population of memory cells that persist in non-lymphoid tissues (NLTs) long after the resolution of an immune response (10–14). They develop *in situ* from T cells that enter an inflamed tissue during the effector stage of an immune response (15). In animal models of virus infection, the establishment of T_RM_ cells is controlled by regulatory T cells (16, 17). It has also been shown in mice that T_RM_ cells respond more rapidly than circulating central memory T cells to the local reoccurrence of a pathogen (14). The binding of αEβ7 integrin heterodimers to E-cadherin on epithelial cells is thought to contribute to the retention of T_RM_ cells in NLTs (18), although not all T_RM_ cells express CD103 (19). αEβ7 integrin is also involved in the maturation of the immunological synapse and promotes the polarization of cytotoxic T cells (20). The potential significance of T_RM_ cells in RE brain is discussed.

## Materials and Methods

All of the surgical specimens used in this study were obtained under IRB approval (UCLA IRB nos. 11-00030 and 13-001213), and with informed consent. In accordance with HIPAA guidelines, all specimens and patient data were de-identified. There were no exclusion criteria. The clinical information for five of the seven RE cases and for four of the eight focal cortical dysplasia (FCD) cases in the present study has been previously published (21). The data for all of the cases are provided in Table S1 in Supplementary Material.

### Flow Cytometry

The isolation and cryopreservation of the brain-infiltrating lymphocytes (BILs) have been previously described (21). In brief, fresh brain tissue was finely minced in dissociation solution (HBSS with 20 mM HEPES pH7.0, 5 mM glucose, and 50 U/ml penicillin/streptomycin), then digested overnight at room temperature in dissociation solution containing 0.5 mg/ml Type IV collagenase (Worthington Biochemical Corp., Lakewod, NJ, USA) and 5% filtered human serum (Mediatech Inc., Manassas, VA, USA). BILs were obtained by fractionation on a 30%: 70% Percoll^®^ (SigmaAldrich, St. Louis, MO, USA) step gradient in RPMI containing 20 mM HEPES. BILs were stained with the following antibodies: APC-efluor^®^ 780-conjugated CD3 (clone UCHT1; eBioscience Inc., San Diego, CA, USA), PE/Cy7-conjugated CD4 (clone SK3; eBioscience Inc.), PerCP/Cy5.5-conjugated CD8 (clone RPA-T8; eBioscience Inc.), APC-conjugated TCR αβ (clone IP26; eBioscience Inc.), FITC-conjugated TCR γδ (clone B1.1; eBioscience Inc), and PE-conjugated CD103 (clone B-Ly7; eBioscience Inc). Data were acquired on an analytical LSRII flow cytometer (Becton Dickinson, San Jose, CA, USA), and were analyzed with FlowJo software (TreeStar Inc., Ashland, OR, USA); histograms were exported into CorelDrawX6 (Corel Corporation, Ottawa, ON, Canada). Statistical analysis and graphing utilized R-project programs (www.r-project.org).

### Immunocytochemistry

Serial sections (5 μm) of paraffin-embedded involved tissue were deparaffinized; antigen retrieval was accomplished by microwaving for 20 min in buffered citrate (10 mM, pH 6.0). Sections were blocked for 1 h (Impress Kit, Vector Laboratories, Burlingame, CA, USA), then incubated overnight at 4°C with a rabbit anti-human CD3 polyclonal antibody (1:800, Dako North America, Inc., Carpinteria, CA, USA) or a rabbit anti-human CD103 monoclonal antibody (clone EPR4166, 1:500, Abcam, Cambridge, MA, USA). Sections were subsequently incubated with a peroxidase-conjugated anti-rabbit secondary antibody (1: 300, Impress Kit, Vector Laboratories) for 1 h at room temperature. Staining was visualized by adding 3, 3′-diaminobenzidine (DAB) (MP Biomedicals, Santa Ana, CA, USA), followed by counterstaining with hematoxylin. Images were acquired using an Aperio ScanScope XT scanner (Aperio, Vista, CA, USA), then transferred to CorelDRAWX6 (Corel Corporation). For immunofluorescence microscopy, fixed free-floating 30 μm cryostat-cut sections were blocked in PBS with 5% normal goat serum (Vector Laboratories) and 0.3% Triton X-100 for 1 h, then incubated in mouse anti-human CD8 (clone C8/144B, 1: 100, Dako) and anti-human CD103 monoclonal antibody (clone EPR4166, 1:500, Abcam) overnight at 4°C followed by incubation in Alexa Fluor^®^ 488 goat anti-rabbit and Alexa Fluor^®^ 568 goat anti-mouse secondary antibodies (1:1000, Life Technologies, Carlsbad, CA, USA) for 1 h at room temperature. Sections were mounted in ProLong^®^ Gold anti-fade reagent containing DAPI (Life Technologies). Images were acquired using an Olympus spinning disk confocal microscope (Olympus America, Inc., Center Valley, PA, USA) under the control of SlideBook™ image acquisition and analysis software (Intelligent Imaging Innovations, Inc., Denver, CO, USA), then transferred to CorelDRAWX6 (Corel Corporation).

## Results

**Figure 1:**
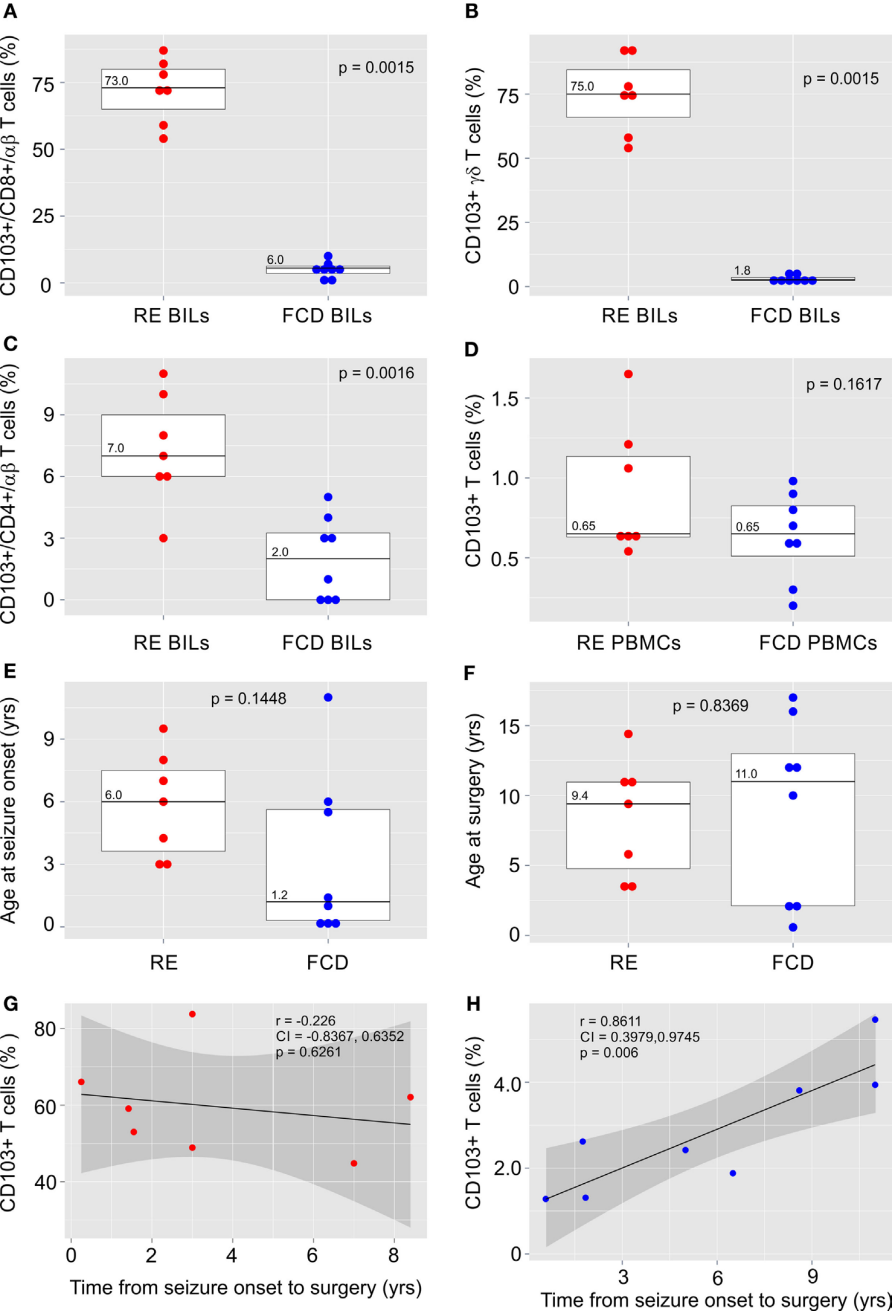
**CD103 expression by T cells isolated from RE and FCD brain specimens**. Box plots with median values showing the percent of CD8^+^ αβ T cells **(A)**, γδ T cells **(B)** and CD4^+^ αβ T cells **(C)** in brain-infiltrating lymphocytes (BILs) that are CD103^+^, and **(D)** the percent of T cells that express CD103 in peripheral blood lymphocytes (PBMCs) from the same patients. In **(E,F)** box plots with median values of patient ages at seizure onset and at surgery are shown. Red dots correspond to individual RE cases (*n* = 7), and blue dots correspond to individual FCD cases (*n* = 8). Calculated *p*-values (Mann–Whitney test in A and B and unequal variance *t*-test in C–F) indicated that there was a significant difference in the relative number of CD103^+^ T cells in RE BILs compared with FCD BILs, but not in peripheral blood. There was no statistical difference between the FCD cases and the RE cases with respect to the age of seizure onset and age at surgery. The linear correlation between the percent of CD103^+^ CD3^+^ T cells in lymphocytes isolated from fresh RE and FCD brain tissue and the length of time between seizure onset and surgery was calculated **(G,H)**, and showed a positive correlation between the relative number of CD103+ T cells in FCD BILs and the length of time between seizure onset and surgery. Pearson correlation coefficients, *p*-values and 95% confidence limits (shaded areas) are shown.

The clinical details of the surgical cases in this study are provided in Table S1 in Supplementary Material. BILs previously isolated from five RE brain surgery specimens (21) and from two new RE cases and eight FCD cases were stained for the T_RM_ cell marker, CD103 (αE integrin subunit) together with antibodies for CD3, CD4, CD8, TCR αβ, and TCR γδ. Inflammatory markers and T cells have been identified in sections of resected brain tissue from FCD surgeries (22), although, to our knowledge, T cells have not been previously isolated from fresh FCD surgical material. The profile of T cell subtypes from the FCD brain specimens varied, although CD8^+^ αβ T cells and γδ T cells predominated, which we also found to be case in RE (21) (Figure S1 in Supplementary Material). As shown in Figures 1A,B, over half of the CD8^+^ αβ T cells and γδ T cells in the RE BIL fractions expressed CD103. There were far fewer CD103^+^ CD4^+^ T cells (Figure 1C) consonant with the observation that few CD4^+^ T cells are found in brain parenchyma of RE patients (7, 9, 23). The percent of CD103^+^ T cells in the BIL fractions from the FCD cases was significantly lower compared with that of the RE cases (Figures 1A–C), whereas the percent of CD103^+^ T cells in the blood of both FCD and RE cases at the time of surgery was very low and not statistically different (Figure 1D).

There was no statistical difference between the FCD cases and the RE cases with respect to the age of seizure onset and age at surgery (Figures 1E,F); however, in marked contrast to the RE cases (Figure 1G), the percent of CD103^+^ T cells was significantly positively correlated with the length of time from seizure onset to surgery, although the relative number of T_RM_ cells remained low (Figure 1H).

The early T cell activation marker, CD69, is also expressed by T_RM_ cells (12). We previously evaluated CD69 expression by flow cytometry and immunocytochemistry to determine whether T cells in RE brain tissue were activated (21). There was good agreement between the percent of CD103^+^ and CD69^+^ T cell subtypes in the BIL fractions from cases RECP32, RECP33, and RECP34 suggesting that all of the CD69^+^ T cells may be T_RM_ cells. For cases RECP26 and RECP37, fewer αβ and γδ T cells were CD69^+^ (21).

**Figure 2:**
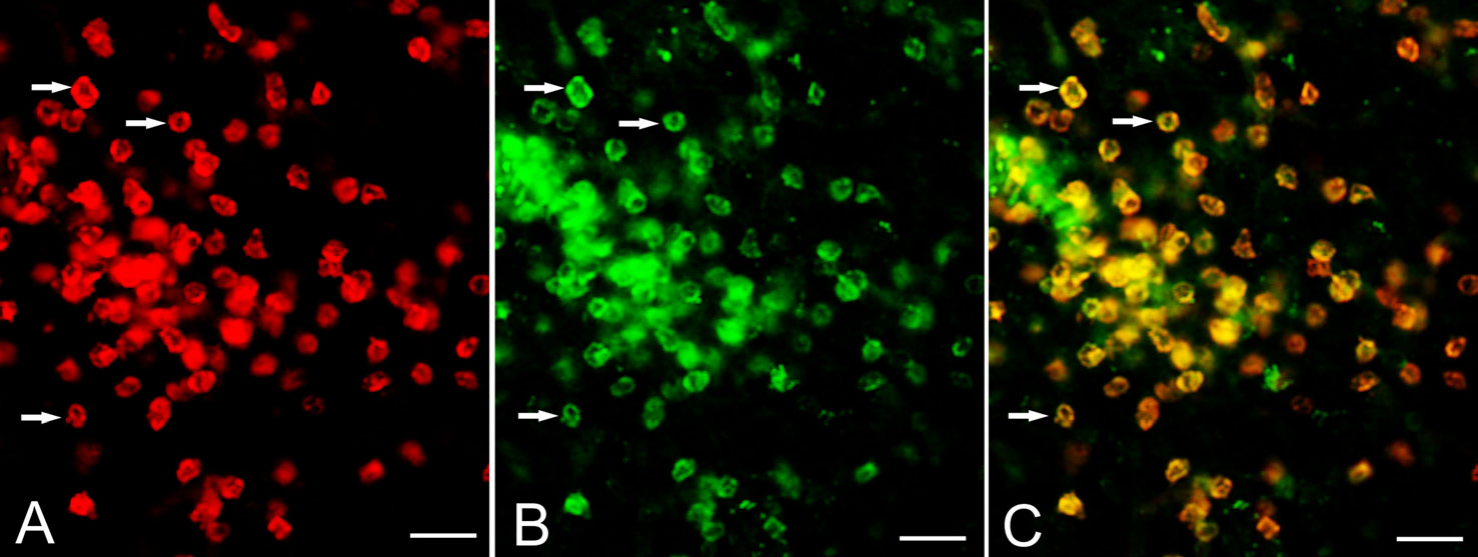
**Expression of CD103 by CD8^+^ T cells**. Cryostat sections of brain tissue from RE surgery case RECP34 were co-stained with CD8 and CD103 antibodies; immunostaining was visualized with Alexa Fluor^®^ conjugated secondary antibodies. **(A)** CD8^+^ T cells in brain parenchyma, **(B)** CD103^+^ staining of the same T cells **(C)** merged image. Arrows point to examples of CD8^+^ CD103^+^ T cells. Scale bars correspond to 50 μm.

The expression of CD103 by T cells was confirmed by co-staining cryostat sections of resected RE brain tissue with CD8 and CD103 antibodies. Figure 2 shows CD8 and CD103 immunoreactivity associated with the cell surface of the same T cells.

**Figure 3:**
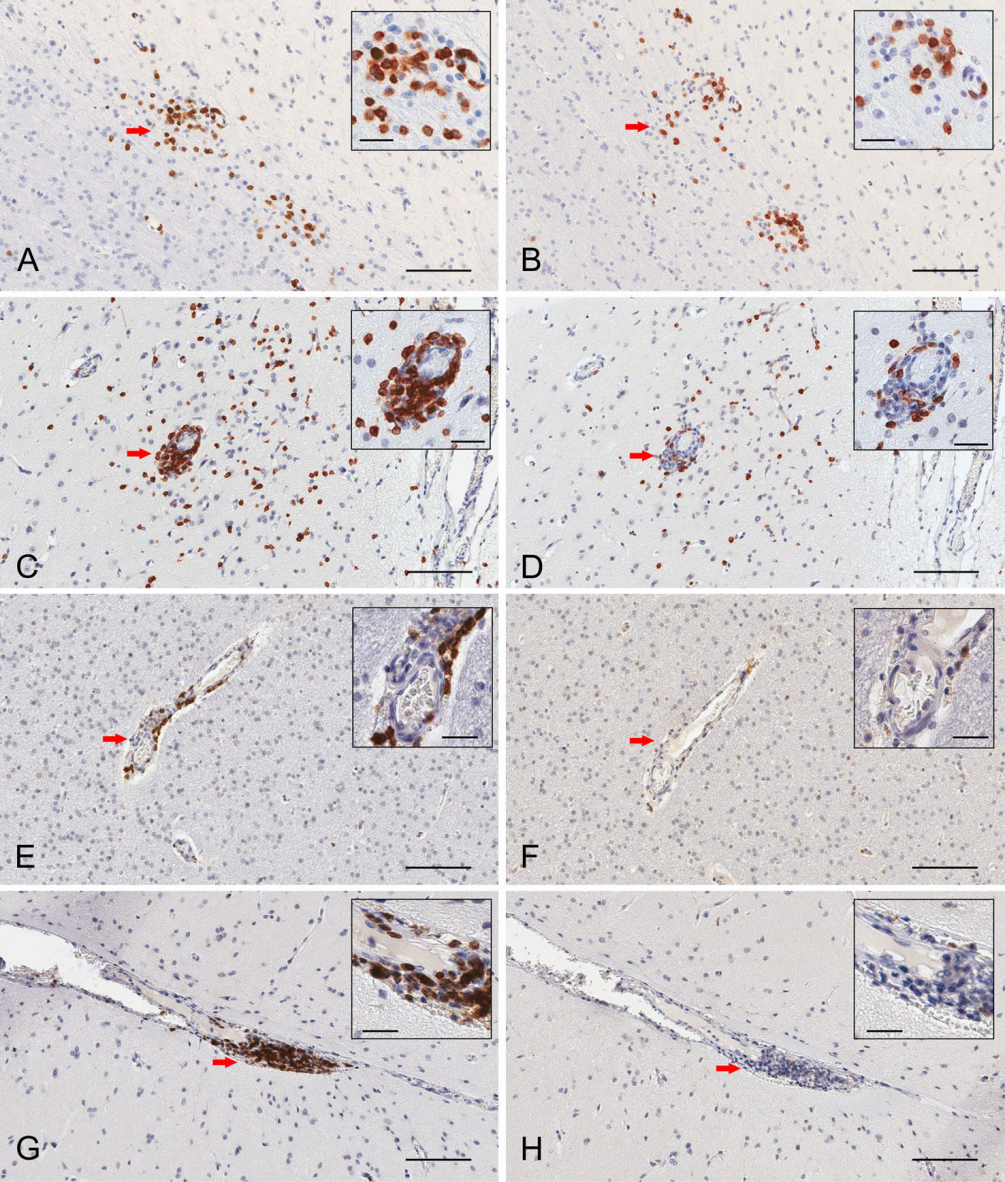
**Immunohistochemical staining of resident memory T cells in RE and FCD patient brain parenchyma**. Serial 5 μm sections of brain tissue from two RE (RECP27 and RECP34) and two FCD surgery cases (CD18 and CD19) were stained with CD3 and CD103 antibodies. Immunostaining was visualized with a peroxidase-conjugated secondary antibody and 3, 3′-diaminobenzidine substrate (brown reaction product). Sections were counterstained with hematoxylin. The same clusters of CD3^+^ T cells **(A)** comprise CD103^+^ cells **(B)**. Scattered T cells in brain parenchyma express CD103 **(C,D)**, but very few T cells in perivascular space are CD103^+^ **(C,D)**. In FCD, CD3^+^ T cells **(E,G)** are confined to perivascular spaces and do not express CD103 **(F,H)**. Insets show magnified views of the areas marked by a red arrow. Scale bars correspond to 100 and 25 μm (insets).

Serial paraffin sections of cerebral cortex from two RE and two FCD surgery cases were also stained with CD103 antibodies. In a representative field of involved RE brain parenchyma, a cluster of CD3^+^ T cells located at the border between white matter and grey matter contains CD103^+^ cells (Figures 3A,B), which are likely to be T cells. Individual T cells that appear to emanate from a blood vessel are also CD103^+^, although far fewer CD103^+^ cells are seen in the perivascular space of the blood vessel (Figures 3C,D). By contrast, in sections of resected brain tissue from the two FCD cases, CD3^+^ T cells are only found in association with blood vessels; few are present in brain parenchyma (Figures 3E,G). The lymphocytes in perivascular spaces do not express CD103 (Figures 3F,H).

## Discussion

Surgery constitutes the last option for the treatment of RE, and is only performed several months to several years after the patient first presents with seizures. If, as is suspected, the disease is triggered by an inflammatory reaction in the brain, we conjectured that some of the T cells found in surgically resected RE tissue may be tissue-resident memory T cells. By flow cytometry, we found that more than half of the CD3^+^ BILs from seven RE surgeries expressed CD103 irrespective of the length of time that had elapsed between the first clinical presentation of the disease and the surgery. Relatively few of the T cells isolated from eight dysplastic brain tissue specimens expressed CD103. The range of ages at the time of surgery and length of time between seizure onset and surgery were similar to the RE cases.

The positive correlation that we observed between the percent of CD103^+^ T cells isolated from FCD brain tissue, and the length of time between seizure onset and surgery indicates that T_RM_ cells may accumulate in the brain over time in cases of FCD, albeit in relatively low numbers, possibly as a consequence of recurrent inflammation caused by the intractable seizures (24). By contrast, the high percentage of T_RM_ cells found in RE brain tissue as early as 3 months after seizure onset is consistent with an acute immune response having occurred at a very early stage of the disease (3, 9).

The clusters of CD103^+^ T cells that we observed in brain parenchyma look remarkably similar to those found in the brains of mice several weeks after an acute intranasal vesicular stomatitis virus infection (25). In this study, it appeared that CD103^+^ T cells were clustered around the original hotspots of viral infection even though no viral transcripts were detected at these sites (25). Other work has shown that T_RM_ cells do not necessarily require the continuous presence of antigen to remain in place (26, 27). Whether an initial inflammatory event in RE is the result of an infection is not known. Cytomegalovirus, herpes simplex virus, and Epstein–Barr virus sequences have been detected in some, but not all, RE brain specimens (28–32). The partially successful treatment of a presumptive RE case with ganciclovir suggests a possible viral etiology (5), as does a patient in Japan who was diagnosed with RE following repeated infection with influenza virus (33). In this case report, the possibility of molecular mimicry as a cause of RE was suggested (33). To investigate whether a persistent viral infection may account for the recurrent immune response in RE, we searched for pathogen-related transcripts in RNA-Seq data from six RE brain tissue samples, but did not find any transcripts to known viruses (unpublished results). In skin, T_RM_ cells are found in psoriatic lesions and sites of fixed drug eruptions (FDEs), and their presence explains the reoccurrence of lesions in the same locations (34). Psoriasis and FDEs have not been linked to known infectious agents, thus, the T_RM_ cells are presumably autoreactive due to a failure of tolerogenic mechanisms, which may also be the case in RE.

In the RE brain tissue examined, it appeared that T cells were actively trafficking into brain parenchyma at the time of surgery (Figure 3C). By contrast, T cells in the FCD brain tissue examined were confined to perivascular spaces (Figures 3E,G), suggesting that the T cells had crossed the endothelial cell barrier, but not the glia limitans. Lymphocyte entry into the brain is a two-step process (35). Following extravasation into perivascular and leptomeningeal spaces T cells must subsequently cross the parenchymal basement membrane and the glia limitans to access the brain (36). In a mouse model of multiple sclerosis, this process involves a complex interplay between cytokines, chemokines, and matrix metalloproteases produced by astrocytes, microglia, non-resident macrophages, and T cells (37–39). Encounter with cells within the perivascular space that present cognate antigen may also be critical for T cell entry into the brain (40).

We have recently shown that identical Vδ1 CDR3 sequences can be detected in RE and FCD brain tissue (21), suggesting that the same γδ T cell clones may traffic to the brain in both diseases, but only enter the brain parenchyma in appreciable numbers in RE. This supposition could be addressed by T cell receptor sequence analysis of CD3^+^ cells isolated by laser capture microscopy from parenchyma and perivascular cuffs in sections of RE and FCD brain tissue. There are several case reports of overlapping RE and FCD pathology (41–46), although none of the cases in the present study were characterized by dual pathology. The presence of T_RM_ cells may explain why T cells can breach the glia limiting in far greater numbers in RE than in FCD. Activation of T_RM_ cells has been shown to recruit circulating T cells into an area of recurrent inflammation (47).

We conclude that the presence of T_RM_ cells suggests that an immune response may precede the clinical presentation of RE, and may account for the difference between RE and FCD with respect to the extent of T cell infiltration into the brain seen at the time of surgery. We speculate that local reactivation of T_RM_ cells, possibly triggered by seizure-induced inflammation (24), recruits antigen-experienced or newly primed T cells into the brain (11), thus perpetuating a chronic inflammatory condition, and progressive destruction of brain tissue. The presence of T_RM_ cells may also explain why, once established, any recurrent immune reaction remains confined to one side of the brain. Treatments designed to block T cell entry into the brain or egress from lymph nodes may not be completely effective if reactivated T_RM_ cells are directly involved in the immunopathology, as was shown to be the case in psoriasis (34).

## Author Contributions

GO designed the study, carried out the flow cytometry, analyzed the data, and drafted the manuscript. JC and MH isolated lymphocytes and performed immunocytochemistry, TC coordinated the collection and processing of surgical specimens, HV provided tissue sections and helped draft the manuscript, and GM provided surgical specimens and helped draft the manuscript.

## Conflict of Interest Statement

The authors declare that the research was conducted in the absence of any commercial or financial relationships that could be construed as a potential conflict of interest.
