# Supplementary material for: Evidence for Resident Memory T Cells in Rasmussen Encephalitis
Source: Front Immunol. 2016 Feb 23;7:64. doi: 10.3389/fimmu.2016.00064 (PMC4763066; doi:10.3389/fimmu.2016.00064)
Supplement: Supplementary file 1 [file Image_1.PDF]

Evidence for resident memory T cells in Rasmussen encephalitis

Geoffrey C. Owens*, Julia W. Chang, My N. Huynh, Thabiso Chirwa, Harry V. Vinters, Gary W. Mathern

*Correspondence: Geoffrey C. Owens: geoffreyowens@mednet.ucla.edu

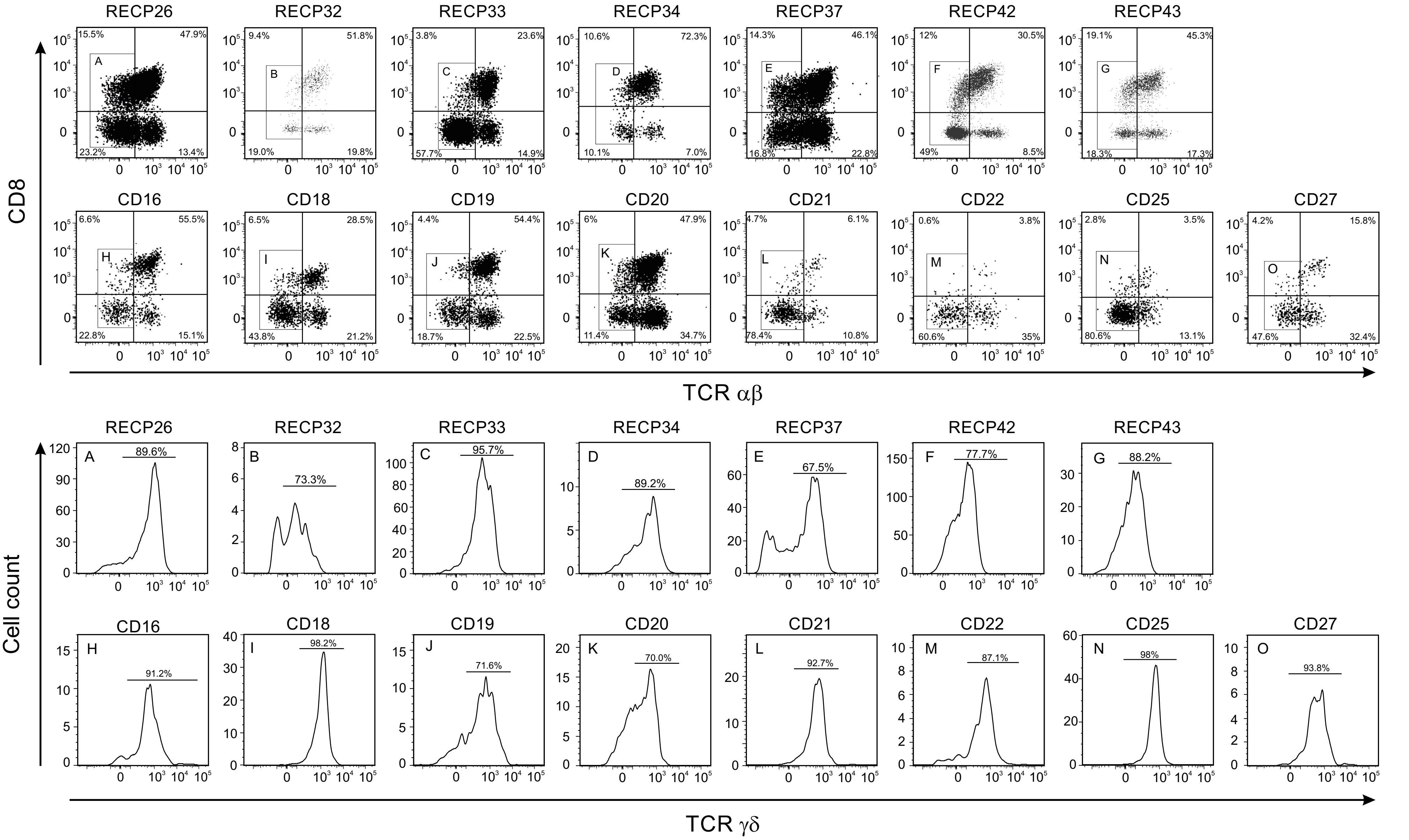

Supplementary Figure 1. Lymphocytes isolated from resected Rasmussen encephalitis and focal dysplasia cortical brain tissue comprise TCR αβ and γδ T cells. Lymphocyte fractions were stained for expression of CD3, CD4, CD8, TCR αβ, TCR γδ, and CD103. $CD3^+$ T cells were first gated on CD8 and TCR αβ; boxed areas (A- O) in the first and fourth quadrants of the 2-D histograms were then gated on TCR γδ. The number of CD3+ T cells gated in each sample was RECP26 11,838; RECP32 929; RECP33 3,824; RECP34 2,027; RECP37 11,839; RECP42 11,256; RECP43 4,020; CD16 1,397; CD18 1,812; CD19 2,809; CD20 5,747; CD21 803; CD22 469; CD25 1,302; CD27 531. RECP, Rasmussen Encephalitis Children's Project; CD, Cortical Dysplasia.
