# Supplementary material for: Evidence for Resident Memory T Cells in Rasmussen Encephalitis
Source: Front Immunol. 2016 Feb 23;7:64. doi: 10.3389/fimmu.2016.00064 (PMC4763066; doi:10.3389/fimmu.2016.00064)
Supplement: Supplementary file 2 [file Table_1.PDF]

Evidence for resident memory T cells in Rasmussen Encephalitis

Geoffrey C. Owens*, Julia W. Chang, My N. Huynh, Thabiso Chirwa, Harry V. Vinters, Gary W. Mathern

***Correspondence:** Geoffrey C. Owens geoffreyowens@mednet.ucla.edu.

Supplementary Table 1: Clinical data from seven Rasmussen encephalitis and eight focal cortical dysplasia patients

CASE ID	ILAE TYPE	GENDER	AGE AT SEIZURE ONSET (YR)	AGE AT SURGERY (YR)	HEMISPHERE
RECP26*		F	6	14.4	L
RECP32*		F	8	11	L
RECP33*		M	4.3	5.8	L
RECP34*		M	9	10.9	R
RECP37*		F	3	3.3	R
RECP42		F	3	3.75	L
RECP43		F	7	9.4	L
CD16*	1c	M	6	17	L
CD18*	1c	M	5.5	12	L
CD19	1c	F	11	16	L
CD20*	1c	M	1.4	10	R
CD21	1b	F	0.25	2	R
CD22	2a	M	0	0.58	L
CD25*	1c	M	0.3	1.1	L
CD27	1c	M	1	12	L

*Previously described
ILAE, International League Against Epilepsy
RECP, Rasmussen Encephalitis Children's Project
CD, Cortical Dysplasia
